# A Modified Intrascleral Intraocular Lens Fixation Technique Using 27-Gauge Blunted Needles with Fewer Intraocular Manipulations

**DOI:** 10.1155/2021/6791977

**Published:** 2021-11-20

**Authors:** Kazuya Morino, Yuto Iida, Masayuki Akimoto

**Affiliations:** Ophthalmology, Osaka Red Cross Hospital, 5-30 Fudegasaki-cho, Tennoji-ku, Osaka 543-8555, Japan

## Abstract

A new method for intraocular lens (IOL) fixation in the scleral tunnel using two common 27G blunted needles and an ultrathin 30G needle with fewer intraocular manipulations was developed. Half-depth scleral flaps were prepared, and vertically angled sclerotomies were performed under each scleral flap, 2 mm from the limbs with a 20G microblade or a 26G needle. Two bent 27G blunted needles connected the sclerotomy and corneoscleral incisions. One haptic was inserted into this bent 27G blunted needle extraocularly and extruded through the sclerotomy site. Each haptic was inserted into the lumen of the preplaced ultrathin 30G needle and buried into the scleral tunnel. In this retrospective study, we reviewed the outcomes of this new technique in patients with at least 3 months' follow-up data. Iris capture of the IOL was not observed in any case, and IOL repositioning was not performed either. Astigmatism induced by intraocular aberration was almost as same as that with other methods. Our technique can be performed in any operation room without any extra instruments. This trial is registered with UMIN000044350.

## 1. Introduction

Implantation of an intraocular lens (IOL) in the absence of capsular support has been accomplished by using an anterior chamber (AC) IOL [1–4], iris-fixed IOL [5–7], and intrascleral fixed posterior chamber (PC) IOL [8–26]. In recent years, sutureless techniques for intrascleral fixation of IOL are gaining popularity among ophthalmic surgeons because of lesser incidence of corneal endothelial cell loss, glaucoma, and peripheral anterior synechiae [1–4], despite the invention of new techniques of retropupillary iris-fixed IOL fixation over the past few years [5–7]. Some studies have shown that corneal endothelial cell density does not significantly change between implantation in AC and retropupillary regions. [6, 7].

**Figure 1:**
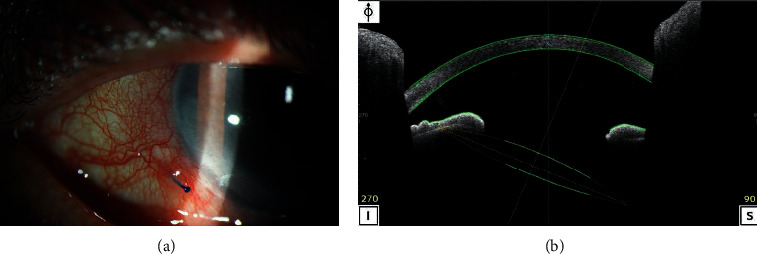
Complications after intrascleral fixation of intraocular lens by the double-needle-flanged technique. (a) Exposure of the flanged haptic and (b) intraocular lens dislocation.

Among the several techniques used for intrascleral fixation of IOL, the double-needle-flanged technique using ultrathin 30G needles and low-temperature cautery is gaining popularity [22]. However, some difficulties in intraocular manipulation, angled sclerotomy, and lens position adjustment without special guidance tools are experienced. Because of these factors, we often observe IOL dislocation or exposure of the flanged haptic, and it is sometimes difficult to reposition the IOL (Figures 1(a) and 1(b)).

Herein, we report a modified method for the intrascleral fixation of IOL, which can be performed extraocularly by navigation of the anatomical markers using two commonly used 27G blunted needles used for viscoelastic material injection and an ultrathin 30G needle. This technique requires fewer intraocular manipulations than the double-needle-flanged technique.

## 2. Materials and Methods

### 2.1. Participants

In this retrospective study, patients who underwent the modified method for intrascleral fixation of IOL between August 2017 and April 2019 were enrolled. Only those patients who had undergone postoperative follow-up examinations for 3 months were selected for this study. The procedures were performed according to the Declaration of Helsinki and were approved by the Osaka Red Cross Hospital ethics committee. Written informed consent was obtained from all patients after the nature and consequences of the study were described to them.

### 2.2. Intraocular Lens

We used a three-piece acrylic IOL (NX-70, Santen Pharmaceutical Co., Ltd, Osaka, Japan.) with an RXJ-70 injection system. The IOL was 13.2 mm long and had a 7 mm diameter optic. The haptics were composed of flexible polyvinylidene fluoride.

### 2.3. Surgical Procedure

A short version of the surgical procedure is shown in Supplementary Video 1.

**Figure 2:**
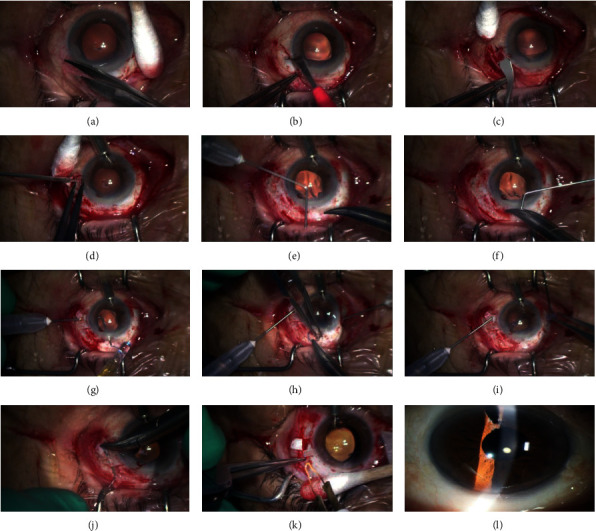
(a) Two-thirds superior conjunctival opening between 4 o'clock and 8 o'clock positions. (b) Half-depth radial scleral groove; (c) half-depth scleral flap; and (d) sclerotomy under each scleral flap using a 20G microblade; (e, f) bent 27G blunted needles (g, h) extruding haptics extraocularly; (i) locking of the trailing haptic with a small silicone piece; (j) burying the haptic into the scleral tunnel using a bent 30G ultrathin needle or (k) flanged haptic in case of a risk of dislocation. (l) A slit-lamp photo of the anterior segment of the eye after the operation.

Under perfusion, the conjunctiva was incised (Figure 2(a)), and a 3.0 mm corneoscleral incision was made at the 11-12 o'clock position using a slit knife. Half-depth radial scleral grooves were made at 3 and 9 o'clock positions (Figure 2(b)). Two more radial grooves were made 2 mm away from each initial groove. Between the two grooves, a half-depth scleral flap was created using a slit knife (Figure 2(c)). After vitrectomy was performed, sclerotomies were made under each scleral flap 2 mm from the limbs using a 20G microblade or 26G needle (Figure 2(d)). Two 27G blunted needles, used for viscoelastic material injection, were bent to connect each sclerotomy site and corneoscleral incision (Figures 2(e) and 2(f)). A bent 27G blunted needle prepared for the leading haptic was inserted into the eye via the sclerotomy site at the 3 o'clock position and was advanced through the surgical corneoscleral incision. The leading IOL haptic was inserted into this bent 27G blunted needle extraocularly (Figure 2(g)). The needle with the leading haptic was pushed into the eye, and the IOL was inserted while the needle sustained the IOL. The needle with the trailing IOL haptic was inserted into the eye through the sclerotomy site at the 9 o'clock position and was advanced through the corneoscleral incision along the optic surface of the IOL (Figure 2(h)). The needle was held with forceps in the left hand. The trailing IOL haptic was inserted into the needle with the needle holder in the right hand. Once the haptic was inserted, the needle was pulled back into the eye and advanced until the trailing haptic was extruded through the sclerotomy site. To avoid unwanted reversion, a small silicone piece was set at the tip of the trailing haptic (Figure 2(i)).

An ultrathin 30G needle was bent at about 4 mm from the tip to introduce each haptic into the scleral tunnels. The bent ultrathin 30G needle was inserted into the sclera parallel to the limbus and between the two grooves that had been made initially. The haptic was inserted into the lumen of the needle and buried into the scleral tunnel (Figure 2(j)). In case of a risk of dislocation, for example, with atopic dermatitis, the ends of haptics were flanged (Figure 2(k)). Finally, peripheral iridotomy was performed using the vitrectomy cutter and the alignment of the IOL was checked.

### 2.4. Astigmatism Induced by Intraocular Aberration

Vector subtraction was performed with refractometer and keratometer measurements to assess intraocular aberration 3 months after the surgery.

### 2.5. Intraocular Lens Tilt Measurements

The angle of tilt of the IOL was measured using swept-source optical coherence tomography (SS-1000 CASIA-2; Tomey Co., Nagoya, Japan) 3 months after surgery.

## 3. Results

Twenty eyes of 16 patients, which included four eyes with aphakia, six with IOL displacement, and ten with lens dislocation, were treated using this new intrascleral fixation technique. In all cases of IOL dislocation, the IOLs were replaced because they were single-piece acrylic IOLs. The ends of haptics were flanged in three cases (15%). The mean patient age at the time of surgery was 67.2 ± 14.3 years (range, 38–85 years). Eleven eyes (55.0%) were of men and 9 eyes (45.0%) were of women. The mean axial length was 23.54 ± 1.34 mm. The patient characteristics are shown in Table 1.

A representative image of the anterior segment of the eye after the surgery is shown in Figure 2(l). The astigmatism induced by intraocular aberration was −0.65 ± 0.34 D. The mean (standard deviation (SD)) IOL tilt was 7.5 ± 3.8°. The mean (SD) preoperative and postoperative corneal endothelial cell densities were 2471 ± 666 cells/mm^2^ and 2006 ± 632 cells/mm [2], respectively. The mean (SD) corneal endothelial cell density loss 3 months postoperatively was −11.0 ± 13.8%.

Complications included three cases of vitreous hemorrhage (15.0%), two cases of postoperative hypotony (<6 mmHg) (10.0%), and one case of intraoperative rhegmatogenous retinal detachment (5.0%). All cases of vitreous hemorrhage disappeared within 2 weeks of surgery. Iris captures of the IOL were not observed in any case, and repositioning of the IOL for IOL tilt was also not performed in any case.

## 4. Discussion

The double-needle-flanged technique using 30G thin-wall needles appears quite simple and minimally invasive even without conjunctival incision. [22] However, some difficulties with intraocular manipulation, angled sclerotomy, and adjustment of the lens position are observed. It is not easy to insert the haptic of the IOL into the needle, especially for beginners, because of the narrow intraocular space, and it is also tricky to constantly shorten the haptic while making a flange. Furthermore, it is difficult to perform an angled sclerotomy through the conjunctiva using a 30G thin-wall needle, 2 mm from the limbs, at a good oblique angle, without any special marker (stabilizer). Owing to these factors, we often observe IOL dislocation or exposure of the flanged haptic, and it is sometimes difficult to reposition the IOL. Therefore, many surgeons have been trying to modify the technique for the better [23, 24, 26]. We considered methods to develop a fool proof technique for intrascleral fixation of IOL with fewer intraocular manipulations. Using the double-needle technique as a reference, we altered each step to develop a technique with commonly used instruments, in combination of the other published techniques.

Scleral flaps were created to bury both the sclerotomy site and the haptics exposed between the sclerotomy site and the tunnel [9, 14]. Sclerotomies were made with a 20G MVR perpendicularly to obtain sufficient space for the adjustment of the haptic alignment and to perform consistently with only anatomical markers [25]. The haptics were extruded using long 27G blunted needles to avoid restricted intraocular manipulations, and 30G ultrathin needles were used as a guide for the insertion of the haptics into the right-sized scleral tunnels [16]. Our modified technique is performed extraocularly under the navigation of anatomical markers and with commonly used instruments without specialized devices.

Vertically angled sclerotomies can be performed using a 26G needle. However, the tightness causes difficulty in alignment of the IOL. Matsumura et al. [25] reported that a smaller sclerotomy incision size for intrascleral IOL fixation was associated with greater IOL tilt and IOL astigmatism after surgery.

The scleral tunnels can be made without conjunctival opening and half-depth scleral flaps using two ultrathin 30G needles [20]. In this case, the first 30G needle was inserted into the sclera from the sclerotomy site to form a scleral tunnel, and the second needle was used to push the first needle out.

On comparing the intraocular and extraocular operation techniques, the extraocular operation can be performed with greater flexibility and is relatively easier. In addition, even under restrictions, the risk of IOL falling into the vitreous cavity is reduced if the operation is performed outside the eye. We have developed this IOL intrascleral fixation technique based on the observable benefits of sutured scleral fixation of IOL and of double-needle-flanged technique.

The ends of haptics were flanged in three patients (15%): one with atopic dermatitis and two others who played tennis and golf frequently. To flange haptics, the haptics were inserted into the guiding needle beyond the scleral tunnel. Once the needle was removed, the end of the haptics appeared on the sclera. After the haptics were flanged, the end was pushed back into the scleral tunnel. This additional step is not complicated; nonetheless, in our experience, it is not required for the majority of cases [16, 20].

Surgical outcomes were comparable to those of previously described techniques of intrascleral fixation of IOL [22, 23, 26] (Table 2). Our study did not have a control group, but the IOL position looked acceptable even when our technique was performed using anatomical markers.

Postoperative complications seem similar between our technique and the previously described techniques of intrascleral and retropupillary fixation of IOL [7, 22, 23, 26] (Table 3). Intraoperative rhegmatogenous retinal detachment was seen in one case (5%) treated with our method. The detachment occurred when posterior vitreous detachment was induced during vitrectomy before IOL fixation; therefore, we consider this to be unrelated to our method.

Although our technique requires conjunctival opening, it can be performed in any operation room without extra instruments. We believe that our technique is easy to try for beginners. After some experience with this technique, surgeons can extend their technique without scleral flaps and conjunctival incision (Supplementary Video 2).

There are several limitations to this study. It was a retrospective and single-arm study, and patients were selected arbitrarily instead of consecutively. Therefore, further research is warranted to validate our findings.

## 5. Conclusions

We have developed a new technique for intrascleral IOL fixation that can be performed extraocularly by navigation of the anatomical markers in combination with established techniques. Surgical outcome and postoperative complications were comparable to those of previously described techniques of intrascleral fixation of IOL. Our technique is easy to attempt because it can be performed in any operation room without extra instruments.

## Figures and Tables

**Table 1:** Patient characteristics.

Characteristics	Data
Number of patients	16
Number of eyes	20
Age (years, range)	67.2 ± 14.3 (38–85)
Sex (male/female)	9/7

Preoperative lens condition (number of eyes)	
Aphakia	4
IOL dislocation	6
Lens dislocation	10
	

IOL, intraocular lens.

**Table 2:** Comparison of surgical results with other techniques of intrascleral fixation of intraocular lens.

Surgical results	Our study	Yamane et al. [22] (double-needle technique)	Kelkar et al. [23] (modified Yamane technique)	Ishikawa et al. [26] (modified Yamane technique)
Number of eyes	20	50	63	29
Intraocular lens model	NX-70 (Santen)	NX-70 (Santen)	Not available	NX-70 (Santen)
Refractive difference (D)	−0.65 ± 0.34	−0.41 ± 0.98	−0.75	0.46 ± 0.84
Astigmatism induced by intraocular aberration	−0.53 ± 0.84	Not available	−1.3	Not available
IOL tilt (°)	7.5 ± 3.8	3.83 ± 2.69	Not available	Not available
Endothelial cell density loss (%)	−11.0 ± 13.8	Not available	Not available	−17.2 ± 18.3%
				

IOL, intraocular lens.

**Table 3:** Comparison of complications with other techniques of intrascleral and retropupillary fixation of intraocular lens.

Complications	Our study	Yamane et al. [22] (double-needle technique)	Kelkar et al. [23] (modified Yamane technique)	Ishikawa et al. [26] (modified Yamane technique)	Mora et al. [7] (retropupillary iris-claw IOL fixation)
Number of eyes	20	100	63	29	32
Temporary corneal edema	2 (10)	1 (1)	0		
Vitreous hemorrhage	3 (15)	5 (5)	2 (3.2)	3 (10.3)	
Rhegmatogenous retinal detachment	1 (5)				1 (3)
Temporary hypotony	2 (10)	2 (2)	1 (1.6)	1 (3.4)	
Temporary IOP elevation	0	2 (2)	2 (3.2)		7 (22)
Iris capture	0	8 (100)	1 (1.6)		
Cystoid macular edema	0	1 (1)	1 (1.6)		8 (25)

IOP, intraocular pressure.

## Data Availability

The data used to support the findings of this study are available from the corresponding author upon request.

## Additional Points

Trial registration: UMIN, UMIN000044350, retrospectively registered (https://upload.umin.ac.jp/cgi-bin/ctr/ctr_view_reg.cgi?recptno=R000048780).

## Supplementary Materials

Supplementary Materials Video S1: this video demonstrates a shorter version of the original technique. Video S2: this video demonstrates a modified version of the technique, without a scleral flap and conjunctival incision.
